# Acute Pancreatitis in Children: Retrospective Analysis of 133 Patients

**DOI:** 10.3390/children13030407

**Published:** 2026-03-15

**Authors:** Gamzenur Yalcinkaya, Gonul Caltepe

**Affiliations:** 1 Department of Pediatrics, Ondokuz Mayis University Faculty of Medicine, 55270 Samsun, Turkey; 2 Department of Pediatric Gastroenterology, Ondokuz Mayis University Faculty of Medicine, 55270 Samsun, Turkey; gcaltepe@omu.edu.tr

**Keywords:** child, length of hospital stay, LDH, enteral nutrition, hospitalization

## Abstract

**Highlights:**

**What are the main findings?**

• In pediatric acute pancreatitis, routinely available early laboratory parameters are significantly associated with the length of hospital stay.
• Early inflammatory markers and the initial clinical course may help identify patients at increased risk for prolonged hospitalization.

**What are the implications of the main findings?**

• Early risk stratification based on simple, accessible parameters may facilitate clinical decision-making in pediatric acute pancreatitis.
• Timely identification of low-risk patients may enable optimized monitoring strategies and more efficient utilization of hospital resources.

**Abstract:**

**Background:** This study aimed to evaluate the etiology, clinic and laboratory features of acute pancreatitis (AP) in children retrospectively. We also aimed to determine the effects of clinical, laboratory and radiological markers on length of hospital stay (LOS). **Materials and methods:** This study was conducted of 133 patients diagnosed with AP. Patients were divided into two groups based on LOS: ≤7 days and >7 days. Demographic, clinical, laboratory and radiological parameters, as well as time to initiation of feeding, were analyzed. **Results:** The mean age of patients was 11.2 ± 4.8 years, and 54.1% were male. The most common etiologies were obstructive (30.8%) and idiopathic (29.3%). Drug-induced and traumatic causes were significantly more prevalent in patients with a hospital stay of more than seven days (*p* = 0.001). Radiological findings other than pancreatic edema (peripancreatic fluid, pleural effusion, or ascites) were significantly associated with prolonged LOS (*p* = 0.002). A positive correlation was observed between LDH and LOS (r = 0.253, *p* = 0.031). LOS was significantly shorter in patients who initiated oral feeding within 48 h (*p* < 0.001). **Conclusions:** LOS in pediatric AP is influenced by laboratory parameters, radiological findings, and the timing of feeding initiation. Identifying early prognostic indicators, particularly in the pediatric patient group, may guide individualized management and improve clinical outcomes.

## 1. Introduction

Acute pancreatitis (AP) is an increasingly common inflammatory disease in childhood, with a clinical course ranging from mild forms to multiple organ failure. Pediatric AP shares similar clinical and pathophysiological features with the adult form, but exhibits distinct differences in terms of etiology, prognosis and management strategies. Therefore, identifying disease severity markers in the early stages is crucial for preventing complications and determining appropriate treatment strategies [1,2]. The prognosis for a complete recovery is excellent in cases of acute pancreatitis that are clinically uncomplicated. However, patients with severe AP may develop shock, high fever, jaundice, ascites, pleural effusion and hypocalcemia. The associated mortality rate of approximately 20% is linked to multiple organ dysfunction, acute respiratory distress syndrome (ARDS), disseminated intravascular coagulation (DIC), shock, renal failure, massive gastrointestinal bleeding and systemic inflammatory response syndrome (SIRS), especially in cases involving systemic or widespread intra-abdominal infection [3].

In adults, various scoring systems such as Ranson, Glasgow (Imrie), BISAP, and APACHE-II have been developed to predict disease severity. However, the validity of these scores in the pediatric population is limited, and pediatric-specific prognostic indicators remain unclear [4,5,6]. The present literature suggests an association between laboratory markers of inflammatory response, including C-reactive protein (CRP), white blood cell count (WBC), and lactate dehydrogenase (LDH) levels, and disease activity and clinical course. Although there has been an increase in the number of studies investigating the relationship of these parameters with prognostic indicators, particularly disease severity and length of hospital stay (LOS), the current data are heterogeneous, and this relationship has not yet been fully established in the pediatric population [4,6,7,8].

Conversely, nutritional strategies implemented during the treatment process are also among the important factors determining the course of the disease and the LOS. The conventional approach of withholding oral intake (NPO) for the purpose of achieving pancreatic rest has been the subject of scrutiny in recent years, as evidenced by the findings of randomised and observational studies. Several studies have compared the LOS and the timing of initiation of oral feeding in children with acute pancreatitis [9,10,11,12,13]. However, most of these studies have focused on patients with mild and/or mild-to-moderate disease, and data regarding severe acute pancreatitis remain limited.

The findings suggest that a comprehensive evaluation of clinical and laboratory markers in pediatric acute pancreatitis may be valuable in predicting disease severity in the early stages, creating individualized treatment plans, and optimizing LOS. The objective of this study is to evaluate the effects of biomarkers (WBC, CRP, LDH, etc.) and feeding timing on the LOS in pediatric acute pancreatitis patients followed at Ondokuz Mayis University Faculty of Medicine Hospital, and to determine predictive parameters for clinical management.

## 2. Materials and Methods

### 2.1. Study Design

The present study comprised 133 patients diagnosed with acute pancreatitis and who were subsequently followed up at the Gastroenterology Clinic of Ondokuz Mayis University Faculty of Medicine Children’s Hospital between the years 2010 and 2022. The initial attacks of 16 patients with recurrent pancreatitis were evaluated. A retrospective review of the patients’ demographic characteristics, clinical features, laboratory data, and length of hospital stay was conducted. The analysis incorporated a range of variables, including WBC, CRP, LDH levels, time to initiation of feeding, age, gender, etiology, presence of complications, and other laboratory parameters.

In accordance with the criteria established by the INSPPIRE consensus, the presence of abdominal pain, elevated serum amylase and lipase levels that exceed the upper limit of normal by a factor of at least three, in conjunction with imaging findings that are consistent with AP, and the manifestation of at least two of these criteria, is indicative of AP. The occurrence of at least two AP attacks, interspersed by pain-free periods or the complete resolution of enzyme levels between attacks, is classified as acute recurrent pancreatitis (ARP) [2].

Patients were divided into two groups according to the length of hospital stay: ≤7 days and >7 days. Patients who remained in hospital for a period exceeding seven days were designated as having experienced prolonged hospitalization, and the factors that could influence such an outcome were investigated. As a secondary objective, etiological factors were compared with clinical demographic and clinical data.

### 2.2. Statistical Analysis

Statistical analyses were performed using SPSS for Mac version 26 software. The normality of the variables was examined using visual (histograms and probability plots) and analytical (Kolmogorov–Smirnov/Shapiro–Wilk tests) methods. Descriptive analyses were provided using interquartile range for non-normally distributed variables. The Mann–Whitney U test and the Kruskal–Wallis test (post hoc Mann–Whitney U test) were performed on numerical data that did not show a normal distribution. The Spearman Correlation Test was employed to analyse the correlation of data that did not conform to a normal distribution. Nominal data were presented using cross-tables, and groups were compared using Chi-Square and Fisher’s exact tests, depending on whether there was a difference between groups. In multivariate analysis, the independent predictors for predicting the probable outcome were examined using logistic regression analysis, with the possible factors identified in the previous analyses. Cases exhibiting a Type I error rate below 5% were deemed to be statistically significant.

## 3. Results

### 3.1. Patient Demographics

The present study comprised a total of 133 patients. The initial attacks of 16 patients with recurrent pancreatitis were evaluated. The patient population was predominantly male, with 54.1% of subjects being male (*n* = 72). The mean age of the patients at baseline was 11.2 years (±4.8 years) (9 months–17.9 years).

### 3.2. Etiological Features

The most prevalent etiological factor in patients was obstructive causes, accounting for 30.8% of cases. The subsequent categories were idiopathic causes (29.3%), drug-induced causes (15.0%), trauma (9.8%), and pancreatitis caused by infectious, chronic, and metabolic diseases. One patient developed pancreatitis due to fungal intoxication (see Table 1).

A number of significant differences were identified in the length of hospital stay according to the underlying cause of the condition. Consequently, drug-induced pancreatitis resulted in prolonged hospitalisation when compared with idiopathic pancreatitis (*p* = 0.001). Furthermore, acute pancreatitis patients with traumatic pancreatitis exhibited prolonged hospitalisation durations in comparison to those with idiopathic and obstructive pancreatitis (*p* < 0.005). The analysis of data gathered on patient age and weight parameters, in addition to etiological factors, revealed that drug-induced pancreatitis manifested more frequently in patients under the age of seven years than in those affected by obstructive causes. A similar observation was made in patients diagnosed with drug-induced pancreatitis, who also exhibited low patient weight. One potential explanation for this phenomenon is the presence of low weight, attributable to the patient’s age. Additionally, patients afflicted with drug-induced pancreatitis often exhibit a cachectic state, a consequence of hematological malignancies (Table 2).

### 3.3. Clinical Features

At the time of diagnosis, the median serum amylase level was recorded as 516 IU/L (range 49–5839 U/L), while the median serum lipase level was 1155 IU/L (range 8–24,543 IU/L). The mean WBC count was 11.142 ± 5.550/mm^3^; the CRP value was 38.5 ± 72.8 mg/L; the LDH value was obtained in 54.8% of patients, with a mean value of 301 ± 182 IU/L. The median LOS was seven days (range 2 to 60 days). In 54.8% of cases, the LOS was seven days or less, and in 45.1% it was more than seven days.

The most common symptoms and findings were abdominal pain (88.7%) and nausea/vomiting (77.4%). It was observed that 14.3% of patients exhibited signs of fever. The symptoms and findings were then divided into systematic groups and compared with hospitalisations. Consequently, no substantial correlation was identified with abdominal pain, nausea/vomiting, fever, and other gastrointestinal symptoms (diarrhea, distension, jaundice, etc.) (Table 3). However, the presence of respiratory system symptoms (e.g., chest pain, dyspnea, pleural effusion), cardiovascular system findings (e.g., tachycardia, hypotension), and the presence of ascites alone demonstrated a positive correlation with the LOS days (Table 3).

Ultrasonography was performed in 94% of patients, magnetic resonance imaging/magnetic resonance cholangiography (MRI/MRCP) in 32.3%, computed tomography (CT) in 17.3%, and endoscopic retrograde cholangiography (ERCP) in 7.5%. In the analysis of the imaging findings, the most prevalent radiological abnormality observed in cases of acute pancreatitis was pancreatic edema, characterised by an increase in size and/or a decrease in pancreatic echogenicity, with a prevalence of 45.1%. The presence of peripancreatic fluid and biliary stones/sludge was identified in approximately one-third of patients. Gallbladder sludge (microlithiasis), cholelithiasis, or choledocholithiasis, detected in the gallbladder or bile ducts, were found in 33.1% of patients. Approximately 30.1% of patients exhibited a minor accumulation of free fluid within the pelvis, while ascites was detected less frequently (11.3%). The findings demonstrated no impact on the LOS in patients with pancreatic edema. Furthermore, the LOS was found to be prolonged in patients with peripancreatic fluid/collection, pleural effusion, pelvic free fluid, and ascites findings (Table 3).

Pancreatic necrosis was detected in 7.1% of patients, 7 of whom had acute pancreatitis due to trauma. Two of the patients with pancreatic necrosis had idiopathic causes, while one patient had acute pancreatitis due to L-asparaginase use for acute lymphoblastic leukemia (ALL). All patients with necrosis had a hospital stay of more than seven days, with a median stay of 17.5 days (10–60 days) (Mann–Whitney *p* = 0.000).

The prevalence of ARP was 12% among the patient cohort. Evaluation of the relationship between etiological factors and recurrent pancreatitis revealed recurrence rates of 12.9% in idiopathic cases, 7.4% in obstructive causes, 20% in drug-induced pancreatitis, and 7.7% in trauma related cases. No significant association was found between the LOS and recurrent acute pancreatitis (*p* = 0.690).

### 3.4. Treatment

All patients received inpatient treatment, and intensive care unit (ICU) admission was required in only four cases (3.0%). Among the patients who required ICU admission, one case was idiopathic pancreatitis; two cases were trauma-related (one due to a firearm injury and another due to a fall from height); and one case was drug-induced, associated with epilepsy and polypharmacy. The conservative management protocol that was implemented included the insertion of a nasogastric tube, NPO/intestinal rest, intravenous fluid support, and analgesia. Within the first 48 h, oral feeding was initiated in 28.5% of patients. With regard to LOS, it was observed that 92.1% of patients who initiated oral feeding within 48 h had a hospital stay of seven days or less, while 95% of those who had a hospital stay of more than seven days commenced oral feeding after 48 h (*p* = 0.000).

## 4. Discussion

The etiology of acute pancreatitis in children differs from that in adults [14]. While alcohol and gallstones are the most prevalent causes in adults, children are affected by a considerably broader range of etiologies. Pediatric acute pancreatitis is recognised as a multifactorial condition in which biliary, drug-related, systemic disease, and traumatic causes are predominant, followed by infectious, metabolic, and genetic factors [15,16,17]. In a study by Poddar et al. [18], the reported etiological distribution was idiopathic (52%), traumatic (21%), biliary (10%), infectious (7%), and drug-induced (5.6%). In contrast, Park et al. [15] identified biliary disease as the most common etiology (32.6%). This finding suggests that the distribution of etiological factors may vary according to regional, ethnic and environmental influences. In the present study, the etiological ranking was as follows: obstructive causes (30.8%), idiopathic (29.3%), drug-related (15%), traumatic (9.8%), infectious (6.8%), and systemic/chronic disease (6%). The prevalence of gallstones and biliary sludge as the predominant obstructive causes was identified, accounting for 85% of cases. The results of this study indicate that biliary and obstructive etiologies, in particular, constitute a major etiological group in the pediatric population of our country [16]. Moreover, the relatively high rate of drug-related cases observed in this study underscores the necessity of close monitoring for acute pancreatitis in children with hematological malignancies and those receiving antiepileptic medications.

Recent studies in the literature have reported that the median length of hospital stay for pediatric acute pancreatitis ranges from 3 to 15 days [7,19,20,21]. In the present study, the median length of hospital stay was seven days. Further analysis of hospital stay revealed that patients with drug-induced and trauma-related pancreatitis had a statistically significant increase in hospital stay compared to those with other etiologies (*p* = 0.001). This finding may be attributable to the presence of concomitant systemic diseases in this cohort, which could contribute to prolonged hospital stay. Previous studies have similarly indicated that systemic diseases and epilepsy treatment related complications prolong hospital stay, particularly in cases of drug-induced pancreatitis (e.g., L-asparaginase, valproic acid) [6].

The most prevalent symptoms observed in patients diagnosed with acute pancreatitis are abdominal pain and nausea/vomiting, as consistently reported in numerous studies in the literature [22,23,24]. The present cohort revealed that fever was identified in 24.3% of patients. In this study, the presence of radiological findings other than pancreatic oedema (e.g., peripancreatic fluid, pleural effusion, ascites) was found to be significantly associated with the LOS (*p* = 0.002). While studies conducted in adult populations have demonstrated that radiological abnormalities are associated with prolonged LOS, confirming this relationship in the pediatric population represents a distinctive finding of the present study [25]. Furthermore, the presence of respiratory system symptoms and findings (*p* = 0.002) and cardiovascular system findings (*p* = 0.027) showed a significant correlation with prolonged LOS. These findings may reflect multi-organ involvement in cases of severe pancreatitis, consequently leading to longer hospital stays. Several studies have also reported that persistent tachycardia is associated with an increased LOS [19]. Conversely, gastrointestinal system findings (e.g., diarrhea, abdominal distension, jaundice) did not show a statistically significant correlation with LOS (*p* > 0.05).

Serum amylase and lipase elevations are widely recognised as the most reliable biochemical indicators of acute pancreatitis. However, no correlation has been demonstrated between amylase and lipase levels and the severity of inflammation or clinical prognosis [26]. Furthermore, severe pancreatitis may occur even in the presence of normal serum amylase and lipase levels. In the present study, it was observed that these biochemical markers did not appear to have a significant impact on LOS. In accordance with this finding, the evidence base supporting the use of WBC count, CRP, and LDH in predicting LOS remains limited. Nevertheless, these biochemical markers are generally used to assess the severity of pancreatitis and have been reported to show an indirect correlation with LOS. Among these parameters, LDH is a general indicator of cellular injury; therefore, its elevation is expected in cases of pancreatic necrosis or systemic involvement. This hypothesis is further supported by the findings of several studies which have indicated that LDH levels may serve as a predictor of LOS [8]. In accordance with the findings of preceding studies, a positive correlation was identified between LDH levels and LOS in the present study (R = 0.253, *p* = 0.031). However, no statistically significant relationship was found between WBC count or CRP and LOS.

Collectively, these findings underscore the prospective function of LDH as a prognostic indicator in the context of pediatric acute pancreatitis. Further research is warranted to elucidate the impact of these parameters on LOS through well-designed prospective, randomised controlled studies.

The management of acute pancreatitis is primarily supportive [27]. Intravenous (IV) fluid therapy is the primary component of acute pancreatitis management. It has been hypothesised that fluid resuscitation may help preserve pancreatic microcirculation by correcting hypovolemia, maintaining adequate perfusion, and preventing microthrombus formation. This, in turn, is believed to reduce complications and prevent progression to severe disease [21]. The findings of multiple studies have demonstrated that early oral/enteral nutrition is associated with a reduction in LOS [28,29,30]. In the present study, 28.5% of patients initiated oral feeding within the first 48 h, whereas 71.5% began feeding thereafter. It was observed that 95% of patients with a LOS exceeding seven days initiated oral feeding after 48 h. Although the timing of the initial oral feeding depends on the severity of the underlying disease, delayed initiation of oral feeding may indicate more severe pancreatitis and therefore predict a prolonged longer hospital stay.

The present study is subject to several inherent limitations. Firstly, the single-center, retrospective design of the study limits the ability to establish clear causal relationships. Furthermore, the exclusion of specific laboratory parameters (e.g., LDH) from the analysis for all patients resulted in diminished statistical power. In addition, radiological evaluation was not uniform across all patients; computed tomography was performed in a limited proportion of cases, potentially restricting comprehensive assessment of pancreatic necrosis and peripancreatic complications. Moreover, trauma-related pancreatitis cases may represent a distinct clinical entity, potentially introducing heterogeneity into the cohort. Notwithstanding, the present study constitutes one of the more comprehensive analyses to date, as it concomitantly evaluates clinical, laboratory, and radiological findings in the pediatric population. Consequently, it is well positioned to serve as a foundation for future prospective studies.

## 5. Conclusions

This study uses data from a relatively large group of patients to investigate the association between clinical, laboratory and etiological factors and the length of hospital stay in paediatric acute pancreatitis. It is important to note that prognostic indicators defined in adult patient groups may not be equally valid in the pediatric population; therefore, the identification of age-specific risk markers is important for clinical management. The findings of this study indicate a potential correlation between clinical findings, LDH levels, and oral feeding timing with length of stay. The findings of this study suggest that the implementation of early risk classification and individualised treatment approaches may yield positive outcomes.

## Figures and Tables

**Table 1:** Distribution of etiological factors.

Etiology	N (%)
Obstructive	41 (%30.8)
Bile sludge/gallstones	35
Choledocholithiasis	1
Type II choledochal cyst	1
Choledochocele	1
Compression by intraabdominal mass	1
Compression by duodenal hematoma	1
Annular pancreas	1
Idiopathic	39 (%29.3)
Drug induced	20 (%15)
L-Asparaginase	8
Valproic Acid	6
Phenobarbital	2
Vincristine	1
Trimebutine Maleat intoxication	1
Colchicine intoxication	1
Trauma	13 (%9.8)
Infectious	9 (%6.8)
COVID-19	3
MIS-C	4
Brucella	1
Hydatid cyst	1
Chronic Disease	8 (%6)
FMF	3
UC + FMF	1
JIA + FMF	1
UC	1
Crohn’s disease	1
DM	1
Metabolic Diseases	2 (%1.5)
Hypertriglyceridemia	2
Fungal Intoxication	1 (%0.8)
Total	133
COVID-19: Coronavirus disease—2019	
MIS-C: Multisystem Inflammatory Syndrome in Children with COVID-19	
FMF: Familial Mediterranean Fever	
UC: Ulcerative colitis	
JIA: Juvenile idiopathic arthritis	
DM: Diabetes mellitus	

**Table 2:** Comparison according to etiological factors, including patient demographics, laboratory data, clinical features, and length of hospital stay.

Etiology	Idiopathic	Obstructive	Drug Induced	Trauma	Others	*p*
**Sex,** ***n*** **(%)**						0.063
**Men**	19 (%48.7)	21 (%51.2)	9 (%45.0)	12 (%92.3)	11 (%55.0)	
**Women**	20 (%51.3)	20 (%48.8)	11 (%55.0)	1 (%7.7)	9 (%45.0)	
**Age (years)**	9.94 ± 4.88	12.89 ± 4.98	9.35 ± 5.5	10.70 ± 4.02	12.75 ± 2.96	**0.014 ^a^**
**Weight (kg)**	35.62 ± 18.8	46.71 ± 19.2	28.8 ± 20.0	39.31 ± 17.1	49.45 ± 17.3	**0.001**
Laboratory data *						
**Amylase, IU/L**	612 (417–612)	720 (323–1829)	391 (189–944)	426 (213–1090)	448 (164–614)	**0.044**
**Lipase, IU/L**	1286 (412–3517)	1583 (686–4404)	1175 (306–2823)	811 (528–1473)	753 (246–1605)	0.096
**CRP, mg/L**	42.34 ± 73.0	18.12 ± 38.6	37.16 ± 71.5	40.66 ± 59.9	73.19 ± 116.1	0.219
**WBC, /mm^3^**	11.78 ± 5.2	10.55 ± 4.0	9.42 ± 7.8	14.36 ± 6.4	10.72 ± 4.9	0.156
**LDH, U/L**	266 (215–319)	256 (197–284)	298 (181–449)	244 (186–376)	260 (217–314)	0.914
**Albumin, gr/dL**	4.3 (4.2–4.5)	4.4 (4.1–4.7)	4.1 (3.4–4.6)	3.9 (3.5–4.5)	4.3 (3.9–4.5)	**0.048**
**ALP, IU/L**	169 (125–222)	161 (96–254)	144 (81–201)	179 (121–209)	158 (97–192)	0.648
**Necrosis,** ***n*** **(%)**	2 (% 5.1)	0	1 (%5.0)	7 (%53.8)	0	-
**Recurrent,** ***n*** **(%)**	5 (% 12.8)	3 (%7.3)	4 (%20.0)	1 (%7.7)	3 (%15.0)	0.648
LOS * (days)	5 (3–7)	6 (4–10.5)	9.5 (6.25–14.75)	10 (7.5–33)	8 (6.25–11.75)	**0.000 ^b^**

^a^ Post Hoc: Drug induced < Obstructive (*p* = 0.037); Drug induced < Others (*p* = 0.003). ^b^ Post Hoc: Trauma > Idiopathic (*p* = 0.001) and Obstructive (*p* = 0.026); Drug induced > Idiopathic (*p* = 0.001). *: Values are presented as median (IQR; 25th–75th percentile). kg: kilogram; CRP: C-reactive protein; WBC: white blood cell; LDH: lactate dehydrogenase; ALP: alkaline phosphatase; LOS: Length of hospital stay. Note: Albumin, LDH and ALP data were incomplete; analyses were performed on available cases (Albumin: *n* = 132, LDH: *n* = 73, ALP: *n* = 78).

**Table 3:** Comparison of clinical findings according to length of hospital stay.

	LOS * (Days) Median (IQR)	LOS ≤ 7 *n*: 73 (%54.8)	LOS > 7 *n*: 60 (%45.2)	*p*
**Age**		11.5 ± 5.0	10.9 ± 4.7	0.264 ^a^
**Sex**				0.866 ^b^
Men	7 (5–11)	40 (%54.8)	32 (%53.3)	
Women	7 (4–10)	33 (%45.2)	28 (%46.7)	
**Weight**		42.3 ± 20.3	38.22 ± 19.3	0.288 ^a^
**Etiology**				**0.001 ^b^**
Idiopathic	5 (3–7)	30 (%41.1)	9 (%15.0)	
Obstructive	6 (4–11)	25 (%34.2)	16 (%26.7)	
Drug induced	10 (6–15)	6 (%8.2)	14 (%23.3)	
Trauma	10 (8–33)	3 (%4.1)	10 (%16.7)	
Others		9 (%12.3)	11 (%18.3)	
**Clinical manifestations**				
Abdominal pain	7 (5–10)	68 (%93.2)	50 (%83.3)	0.075 ^b^
Vomiting/Nausea	7 (5–10)	60 (%82.2)	43 (%71.7)	0.148 ^b^
Fever	8 (6–11)	9 (%12.3)	10 (%16.7)	0.477 ^b^
GIS manifestations	7 (5–10)	10 (%13.7)	9 (%15.0)	0.831 ^b^
Respiratory manifestations	14.5 (7.25–25.5)	4 (%5.5)	12 (%20.0)	**0.010 ^b^**
CVS manifestations	11 (8.75–19.5)	2 (%2.7)	8 (%13.3)	**0.021 ^b^**
Ascites	13.5 (8.5–19.75)	2 (%2.7)	8 (%13.3)	**0.021 ^b^**
Laboratory data *				
Amylase, IU/L		532 (89–5283)	508 (49–5839)	0.679 ^a^
Lipase, IU/L		1174 (55–14,050)	1141 (8–24,543)	0.998 ^a^
CRP, mg/L		25.42 ± 45.6	54.57 ± 94.1	0.055 ^a^
WBC, /mm^3^		10.78 ± 5.12	11.56 ± 6.05	0.337 ^a^
LDH, U/L		244 (143–362)	277 (142–1121)	**0.026 ^a^**
**Necrosis**	17.5 (11.5–32.75)	0	10 (%16.7)	**0.000 ^b^**
**Nutrition starting time**				**0.000 ^b^**
≤48 h	4 (3–6)	35 (%47.9)	3 (%5.0)	
>48 h	9 (6–12)	38 (%52.1)	57 (%95.0)	
**Recurrent**	7 (5.25–14)	10 (%13.7)	6 (%10.0)	0.514 ^b^

^a^ Mann–Whitney U test. ^b^ Chi-square test. *: Values are presented as median (IQR; 25th–75th percentile). CRP: C-reactive protein; WBC: white blood cell; LDH: lactate dehydrogenase; LOS: length of hospital stay; GIS: gastrointestinal system; CVS: cardiovascular system.

## Data Availability

Data are available from the corresponding author upon reasonable request. The data are not publicly available due to privacy and ethical restrictions.
